# Identification and characterization of QTLs for brown planthopper resistance from wild rice, *Oryza rufipogon*

**DOI:** 10.1270/jsbbs.25027

**Published:** 2025-11-21

**Authors:** Hoang Nam Nguyen, Takashige Ishii, Sachiyo Sanada-Morimura, Shao-Hui Zheng, Daisuke Fujita

**Affiliations:** 1 The United Graduate School of Agricultural Sciences, Kagoshima University, 1-21-24 Korimoto, Kagoshima 890-0065, Japan; 2 Graduate School of Agricultural Science, Kobe University, 1-1 Rokkodai, Nada, Kobe, Hyogo 657-8501, Japan; 3 Agro-Environment Research Division, Kyushu Okinawa Agricultural Research Center, NARO, 2421 Suya, Koshi, Kumamoto 861-1192, Japan; 4 Faculty of Agriculture, Saga University, 1 Honjo-machi, Saga 840-8502, Japan

**Keywords:** QTL identification, BPH resistance, wild rice, BRIL, *Nilaparvata lugens*

## Abstract

The brown planthopper (*Nilaparvata lugens* (Stål); BPH) is a serious pest of rice (*Oryza sativa* L.). Host plant resistance is an effective means of controlling it; at least 46 BPH resistance genes have been identified. However, BPH can overcome resistance genes, so we need to detect more genes. To find new BPH resistance genes in *Oryza rufipogon*, we analysed quantitative trait loci (QTLs) associated with BPH resistance. Using 161 backcross recombinant inbred lines derived from *O. rufipogon* accession W0630, we identified eight loci associated with resistance. *qBPH5* and *qBPH6*, on chromosomes 5 and 6, were validated in F_2_ and F_2:3_ populations derived from crosses between *O. sativa japonica* variety ‘Nipponbare’ and the inbred lines. BPH resistance of a line carrying both QTLs was higher than that of lines carrying just one. Pyramiding improved resistance and can be used in breeding BPH-resistant rice.

## Introduction

Rice (*Oryza sativa* L.) is an important crop for more than half of the world’s population. However, rice cultivation is compromised by the brown planthopper (BPH), *Nilaparvata lugens* (Stål), a serious insect pest, which feeds directly on the plant, reducing its grain yield. Under high pest density, damage can lead to a condition known as “hopperburn”. The insects also transmit *Rice grassy stunt virus* (RGSV) and *Rice ragged stunt virus* (RRSV), which can further reduce yield (Cabauatan *et al.* 2009). BPH directly caused the loss of 2.7 million tonnes of rice in China, and BPH-transmitted viruses (mainly RGSV and RRSV) reduced Vietnam’s yield by 0.4 million tonnes from 2005 to 2008 (Brar *et al.* 2009, Hu *et al.* 2016). In western and southwestern Japan, economic losses of 10.5 × 10^9^ JPY in rice production were reported in 2013, with similar losses occurring again in 2019 (Sanada-Morimura 2020). BPH is controlled primarily by chemical pesticides, but this approach is both economically and environmentally unsustainable. In addition, insecticides eliminate its natural predators, and the overuse of pesticides disrupts the ecological balance, resulting in the resurgence of more virulent BPH populations (Tanaka *et al.* 2000). Therefore, the use of host plant resistance offers a more economically and environmentally sustainable strategy for managing this pest effectively.

At least 46 genes associated with BPH resistance (designated *BPH1* to *BPH46*) have been identified and mapped in cultivated and wild rice (Fujita *et al.* 2013, Li *et al.* 2024, Yan *et al.* 2023). More than half of these genes originated from wild rice, suggesting a rich genetic diversity and potential for harbouring novel alleles (Brar and Khush 1997).

However, over the years, BPH has monogenic resistant cultivars within a few generations (Tanaka and Matsumura 2000). In 1973, IR26 was released as the first cultivar with *BPH1* resistance and reduced BPH damage for 3 years until it was overcome in 1976 (Khush and Virk 2005). Subsequently, cultivars carrying *BPH2* were widely grown, but a more virulent BPH population then adapted to it (Kobayashi 2016). Cultivars carrying *BPH5*, *BPH7*, *BPH8*, *BPH9*, *BPH10*, and *BPH18* were also overcome by BPH populations across Asia (Horgan *et al.* 2015). More recently, *BPH20*, *BPH21*, *BPH25*, *BPH26*, and *BPH32* have become less effective (Fujii *et al.* 2021, Myint *et al.* 2012, Nguyen *et al.* 2019). These facts indicate the vulnerability of single BPH resistance genes to BPH. Therefore, it is necessary to detect new resistance genes.

Cultivated rice (*O. sativa*) was domesticated from *O. rufipogon*. This makes *O. rufipogon* a good, accessible, compatible wild genetic resource for the improvement of rice (Thanh *et al.* 2011). More BPH resistance genes has been reported from *O. rufipogon* than other wild rice species, including *O. officinalis*, *O. nivara*, *O. minuta*, *O. australiensis*, *O. eichingeri*, and *O. latifolia* (Huang *et al.* 2013, Li *et al.* 2019, Wang *et al.* 2022, Yan *et al.* 2023, Yang *et al.* 2020, Zhang *et al.* 2020). Here, we evaluated BPH resistance among seven *O. rufipogon* accessions and found strong resistance in accession W0630, from Myanmar, to a highly virulent BPH population. To reveal the genetic basis of BPH resistance in W0630, we performed QTL analysis using a set of backcross recombinant inbred lines derived from W0630 in the ‘Nipponbare’ genetic background. We validated and characterized QTLs through progeny testing. The findings will be useful in breeding rice cultivars with resistance to BPH by marker-assisted selection (MAS).

## Materials and Methods

### Plant materials

We screened seven *O. rufipogon* accessions for BPH resistance: W1866 from Thailand, W0630 and W0610 from Myanmar, W0137 and W0107 from India, W1230 from Indonesia, and W1294 from the Philippines. Seeds were provided by the National Institute of Genetics, Japan. To identify QTLs, we used a set of 161 backcross recombinant inbred lines (BRILs) at BC_2_F_8_ derived from a cross between *O. sativa japonica* variety ‘Nipponbare’ and W0630 (with an annual growth habit) (Thanh *et al.* 2011) (Supplemental Fig. 1). The BRILs and the parents were provided by the Laboratory of Plant Breeding, Kobe University, Japan. To confirm QTLs in the BRILs, we used F_2_ populations derived from lines with the least amount of W0630 segments and high BPH resistance. We crossed BRIL16 (with *qBPH1*, *qBPH6*, *qBPH9*, and *qBPH11*), BRIL29 (with *qBPH2* and *qBPH5*) and BRIL176 (with *qBPH12.1* and *qBPH12.2)* (Supplemental Fig. 2) with ‘Nipponbare’ to create F_1_ plants. We genotyped F_2_ populations of each cross and evaluated F_2:3_ populations for BPH resistance.

To evaluate the effects of *qBPH5*, *qBPH6*, and their combination, three lines were selected. A line carrying *qBPH5* was selected from the F_3_ population derived from BRIL29 × ‘Nipponbare’ and a line carrying *qBPH6* was selected from the F_3_ population derived from BRIL16 × ‘Nipponbare’. A line carrying both *qBPH5* and *qBPH6* was directly selected among the 161 BRILs. All three lines relatively had high MSST damage scores and minimal background segments from W0630, allowing for focused evaluation of the QTL effects. The lines carrying QTLs were selected using SSR markers flanking the QTL regions.

### DNA extraction and genotyping

Total DNA from BRILs and F_2_ populations was extracted by the potassium acetate method (Dellaporta *et al.* 1983). The 161 BRILs were genotyped using 180 SSR markers as described by Thanh *et al.* (2011). We genotyped 150 F_2_ plants of each population with SSR markers polymorphic between the parents (McCouch *et al.* 2002, Temnykh *et al.* 2001). Polymerase chain reaction (PCR) was performed to determine the genotypes of F_2_ plants with SSR markers. The PCR amplification mixture (8 μL) consisted of 3 μL of 2X GoTaq Green Master Mix (pH 8.5), 1 μL of 0.25 μM primers, and 4 μL of 1:20-diluted DNA sample. The PCR protocol comprised initial denaturation at 96°C for 5 min; 35 cycles of 96°C for 30 s, 55°C for 30 s, and 72°C for 30 s; and a final extension at 25°C for 1 min. The PCR products were electrophoresed in 4% agarose gel, stained with ethidium bromide in 0.5 × TBE buffer for 1 h, and visualized under ultraviolet light.

### BPH populations for resistance evaluation

We evaluated BPH resistance with two BPH populations, Hadano-1966 and Koshi-2013. Hadano-1966 (collected from Hadano City, Kanagawa Prefecture, Japan, in 1966) was captured before any BPH resistance genes were released and has weak virulence. Koshi-2013 (collected from Koshi City, Kumamoto Prefecture, Japan, in 2013) overcame *BPH1* and *BPH2* (Fujii *et al.* 2021). Both populations were maintained on the susceptible *japonica* ‘Reiho’ at 25°C under 16-h light / 8-h dark at the National Agriculture and Food Research Organization. Both have been transferred to Saga University and maintained on ‘Taichung 65’ (T65) for use in our experiments at Saga University under the same conditions. We used T65 because it is commonly used in our lab as susceptible check variety in BPH resistance studies, rearing on T65 helping maintain consistency in the resistance evaluation.

### Modified seedbox screening test

To evaluate BPH resistance in BRILs and F_2:3_ populations, we used the Hadano-1966 BPH population in a modified seedbox screening test (MSST) (Horgan *et al.* 2015) at 25°C. In a plastic tray measuring 23.0 cm × 30.0 cm × 2.5 cm, we sowed 25 seeds of each line in a single row with three rows of ‘Nipponbare’ and one row of the resistant donor parent (2.5 cm between rows), with row positions randomized. At 7 days after sowing, plants were thinned to 20 per row and infested with second and third instar nymphs at the rate of 20 nymphs per plant. When all susceptible parents had died, the damage score of plants was determined according to the standard evaluation scoring system for rice (IRRI 2014).

### Antibiosis test

We performed antibiosis tests (described by Tanaka 2000) to investigate for BPH resistance among the wild rice accessions. Seven *O. rufipogon* accessions and control lines were independently sown in 215-mL plastic cups with five replications. Thirty-day-old plants were covered with a transparent plastic cup and infested with 5 thin-abdomen brachypterous female BPHs. At 5 days after infestation (DAI), the percentage of dead BPH was calculated and used as adult mortality.

### Antixenosis test

One plant each of a line with chromosomal segments of W0630 covering the QTLs and ‘Nipponbare’ were planted together in a 215-mL plastic cup with 5 replications. At 30 days of age, the plants were enclosed in plastic tubes equipped with ventilators. Inside each tube, we released 20 second instar nymphs. In this paired design, BPH could freely choose between the two plants, allowing the evaluation of settling preference. In addition, a separate cup with W0630 and ‘Nipponbare’ was used as a resistant–susceptible control. The number of insects settled on each plant was recorded daily until 5 DAI. The percentage of insects that settled on each plant determined the level of antixenosis (Nguyen *et al.* 2021).

### QTL analysis

We performed QTL analysis of BRILs and F_2_ populations using their genotypic and phenotypic data. In Windows QTL Cartographer v. 2.5 software, QTLs were estimated by single-marker analysis, interval mapping (IM), and composite interval mapping (CIM) methods (Wang *et al.* 2012). The optimal logarithm of odds (LOD) threshold was used to determine the presence of QTLs at threshold values of 2.5 for BRILs, 2.6 for BRIL16/ ‘Nipponbare’ F_2_, and 2.1 for BRIL29/‘Nipponbare’ F_2_. The percentage of phenotypic variance explained (PVE) by the QTLs and the additive effect were estimated by the software.

### Statistical analysis

The mean values of BPH resistance of lines carrying QTLs were compared by one-way ANOVA. Dunnett’s test and Tukey–Kramer analysis were used for multiple comparisons of damage score, adult mortality, and antixenosis levels of lines carrying QTLs in R v. 4.3.2 software.

## Results

### QTL detection for BPH resistance using BRILs

**Fig. 1.**
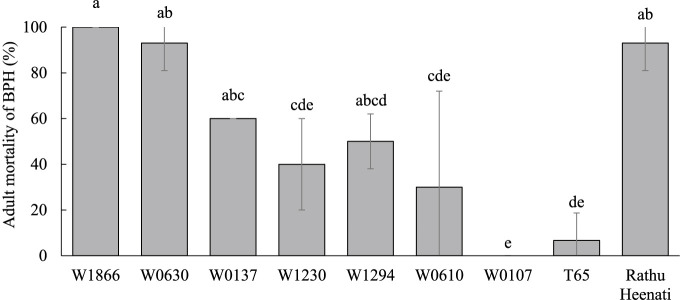
Adult mortality (%) of brown planthopper (BPH) on seven *O. rufipogon* accessions, ‘Rathu Heenati’ (resistant) and ‘Taichung 65’ (T65) (susceptible) infested by Koshi-2013 at 5 days after infestation. Bars with the same letter are not significantly different at *P* < 0.05 by Tukey–Kramer test.

**Fig. 2.**
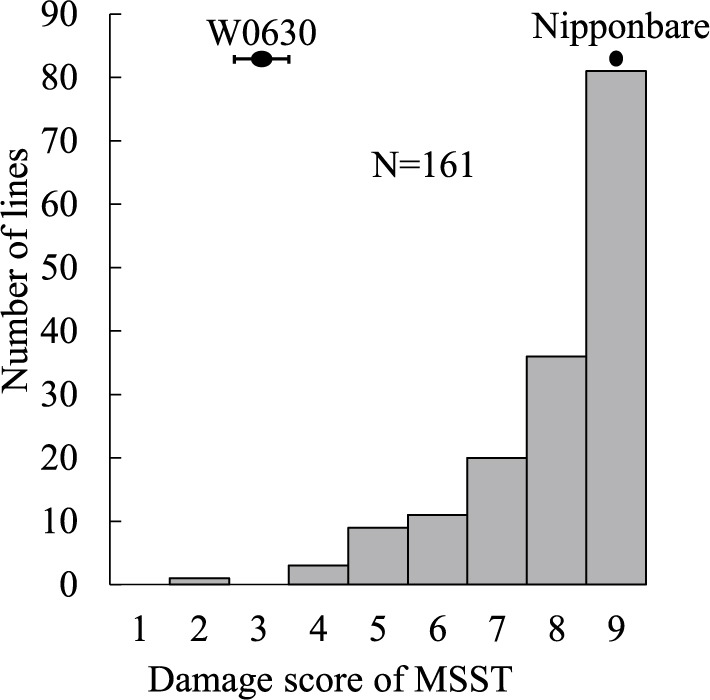
Frequency distribution of damage score in modified seedbox screening test (MSST) in ‘Nipponbare’ × W0630 BRILs infested by Hadano-1966. Bars indicate means in parents with standard deviation.

We screened seven accessions of *O. rufipogon* for BPH resistance. W0630 and W1866 had strong resistance to Koshi-2013 in the antibiosis test (Fig. 1). The MSST damage score of W0630 was 3.75, while that of the susceptible ‘Nipponbare’ was 9.0 (Fig. 2). To identify the genetic basis of BPH resistance in W0630, we evaluated the 161 BRILs derived from W0630 by MSST. The damage scores ranged from 2 to 9, with a continuous frequency distribution, suggesting that BPH resistance in W0630 is controlled by multiple loci (Fig. 2). Single-marker analysis identified eight QTLs for BPH resistance across seven chromosomes: *qBPH1* (linked to marker RM243 at 7.97 Mb) on chromosome (chr.) 1, *qBPH2* (RM263 at 25.89 Mb) on chr. 2, *qBPH5* (RM122 at 0.28 Mb) on chr. 5, *qBPH6* (RM587 at 2.29 Mb) on chr. 6, *qBPH9* (RM410 at 17.59 Mb) on chr. 9, *qBPH11* (RM167 at 4.06 Mb) on chr. 11, *qBPH12.1* (RM247 at 3.19 Mb) on chr. 12, and *qBPH12.2* (RM309 at 21.52 Mb) on chr. 12. The W0630 alleles at all QTLs increased BPH resistance (Table 1). Interval mapping identified six of these QTLs (except *qBPH5* and *qBPH11*), and uniquely identified *qBPH6* (PVE = 8.2%) and *qBPH9* (15.6%); and both IM and CIM detected the other four: *qBPH1* (PVE = 19.8%, IM; 7.3%, CIM) on chr. 1, *qBPH2* (PVE = 13.1%, IM; 9.4%, CIM) on chr. 2, *qBPH12.1* (PVE = 11.3%, IM; 8.4%, CIM) on the short arm of chr. 12, and *qBPH12.1* (PVE = 7.7%, IM; 5.9%, CIM) on the long arm of chr. 12 (Table 2). Although the peak LOD positions of *qBPH2* identified by IM and CIM were slightly different, the regions with LOD scores above the threshold overlapped broadly (IM: 20.41–29.59 Mb; CIM: 24.04–29.59 Mb), indicating that both models detected the same QTL region. The W0630 alleles at all detected QTLs reduced the damage score and increased BPH resistance.

### QTL confirmation for BPH resistance in F_2_ and F_2:3_ populations

**Fig. 3.**
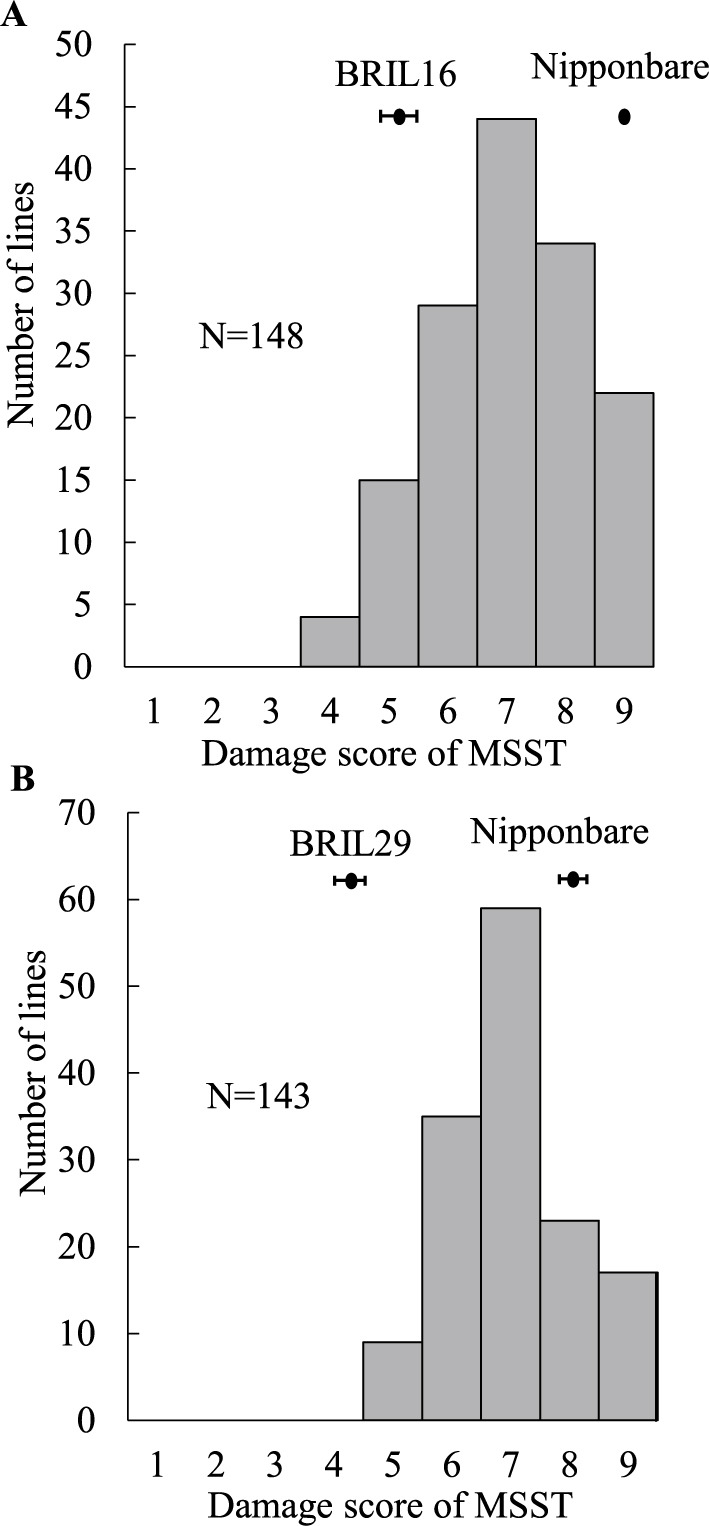
Frequency distribution of damage score in modified seedbox screening test (MSST) in the (A) BRIL16 × ‘Nipponbare’ and (B) BRIL29 × ‘Nipponbare’ F_2:3_ populations infested by Hadano-1966. Bars indicate means in parents with standard deviation.

To confirm QTLs for BPH resistance, we conducted QTL analysis of the F_2:3_ population derived from ‘Nipponbare’ × BRILs containing the W0630 chromosomal segment with the detected QTL regions (BRIL16, BRIL29, and BRIL176). Those lines were selected based on their low damage score in MSST compared to other BRILs in Fig. 2 (BRIL16 = 4, BRIL29 = 4, and BRIL176 = 5). The damage score of BRIL16 was 5.5, that of ‘Nipponbare’ was 9.0 and those of the F_2:3_ population ranged from 4 to 9 (Fig. 3A). We detected two QTLs for BPH resistance: *qBPH1* (between RM23 and RM5638) on chr. 1 and *qBPH6* (between RM589 and RM204) on chr. 6; both W0630 alleles increased BPH resistance (Table 3). Single-marker analysis showed that BRIL16 has chromosomal segments from W0630 with *qBPH1*, *qBPH6*, *qBPH9*, and *qBPH11* (Supplemental Fig. 2), but CIM confirmed only *qBPH1* and *qBPH6*.

The damage score of BRIL29 was 4.8, that of ‘Nipponbare’ was 8.2, and those of the F_2:3_ population ranged from 5 to 9 (Fig. 3B). QTL analysis identified a single QTL, *qBPH5*, between RM7029 and RM1024 on chr. 5 (PVE = 11.74%); the W0630 allele increased BPH resistance (Table 4). Single-marker analysis showed that BRIL29 has chromosomal segments from W0630 with *qBPH2* and *qBPH5* (Supplemental Fig. 2); IM confirmed only *qBPH5*. The damage score of BRIL176 was 6.0, that of ‘Nipponbare’ was 9.0, and those of the F_2:3_ population ranged from 4 to 9 (Supplemental Fig. 3). Single-marker analysis showed that BRIL176 has chromosomal segments from W0630 with *qBPH12.1* and *qBPH12.2* (Supplemental Fig. 2). However, we did not detect any QTLs.

### Characterization of QTLs for BPH resistance

**Fig. 4.**
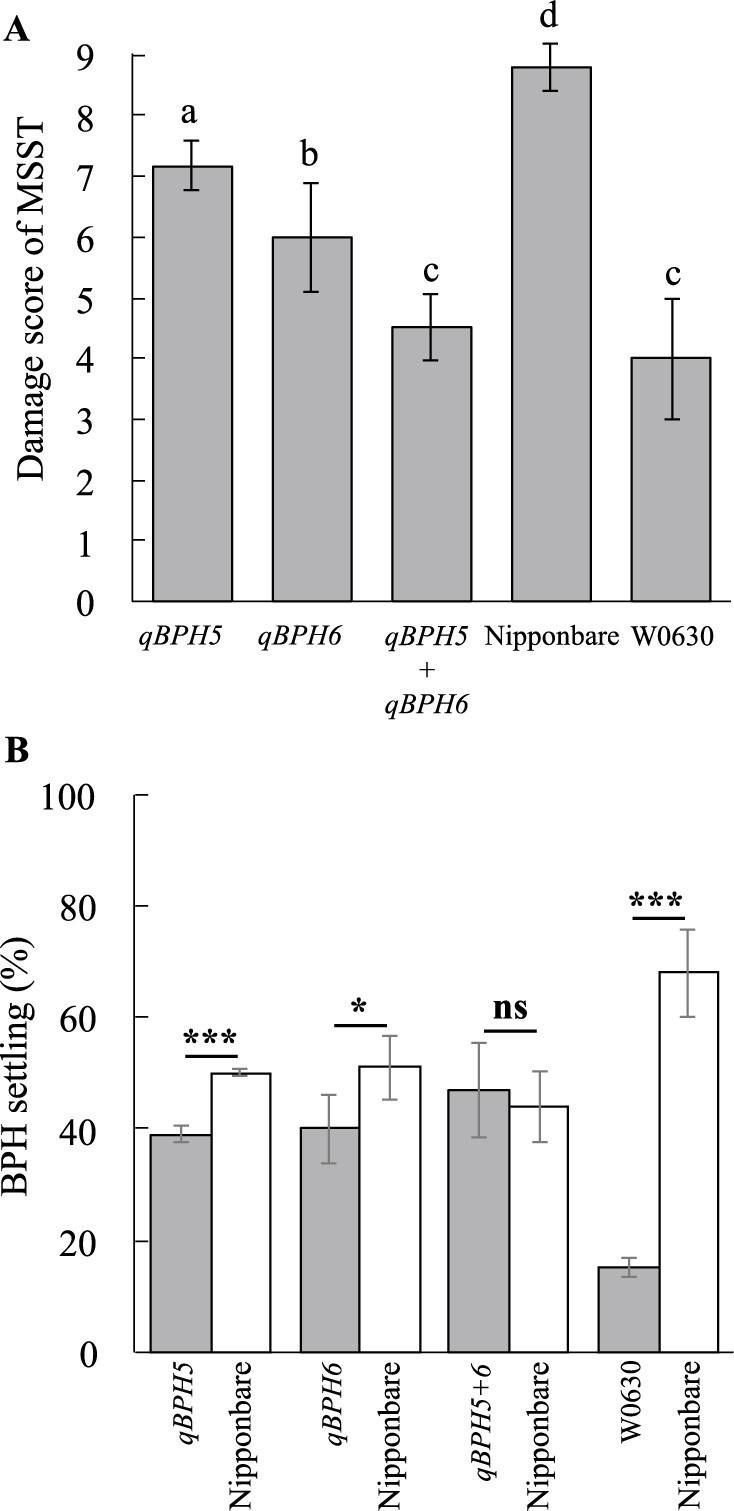
Effects of *qBPH5*, *qBPH6*, and their pyramiding on (A) damage score in modified seedbox screening test (MSST) and (B) antixenosis test using Hadano-1966 BPH population. Bars indicate standard deviation. (A) Bars with the same letter are not significantly different between genotypes by Tukey–Kramer multiple comparison test (*P* < 0.05). (B) Asterisks indicate significant difference between the indicated line and ‘Nipponbare’: *P < 0.05, ***P < 0.001 by *t*-test.

To evaluate the resistance conferred by and the effect of the identified QTLs, we conducted MSST using the Hadano-1966 BPH population. The damage scores of lines carrying *qBPH5* and *qBPH6* were 7.2 and 6.0, respectively, while that of a line carrying both QTLs was 4.5, significantly lower than that of either alone and nearly equivalent to that of W0630 (4.0). These damage scores were significantly lower than that of the susceptible ‘Nipponbare’ (8.8) (Fig. 4A). In the antixenosis test, 39% of BPH settled on the line carrying *qBPH5*, significantly less than the 50% on ‘Nipponbare’; 40% settled on the line carrying *qBPH6*, significantly less than the 51% on ‘Nipponbare’; and 47% settled on the line carrying *qBPH5* and *qBPH6*, similar to the 44% on ‘Nipponbare’; 15% settled on W0630, significantly less than the 68% on ‘Nipponbare’ (Fig. 4B).

## Discussion

At least 46 BPH resistance genes have been identified (Fujita *et al.* 2013, Li *et al.* 2024, Yan *et al.* 2023). Here, we identified several QTLs associated with BPH resistance on chrs. 1, 2, 5, 6, 9, 11, and 12 by using BRILs (Tables 1, 2), and validated *qBPH5* and *qBPH6* in F_2_ populations. Several BPH resistance genes have been identified on the short arm of chr. 6: *BPH3* in ‘Rathu Heenati’ (Jairin *et al.* 2007), *BPH4* in ‘Babawee’ (Kawaguchi *et al.* 2001), *BPH25* in ‘ADR52’ (genetically distinct from *BPH3* and *BPH4*) (Myint *et al.* 2012), *BPH29* in ‘RBPH54’, an introgression line derived from *O. rufipogon* (Wang *et al.* 2015), and *BPH32* in Ptb33 (Ren *et al.* 2016). In the same region we identified *qBPH6* between RM589 (1.38 Mb) and RM204 (3.17 Mb) (Table 3). This region overlaps with *BPH4* (RM589–RM586, 1.38–1.48 Mb) and *BPH25* (S00310–RM8101, 0.21–1.7 Mb). *qBPH6* also partially overlaps with *BPH3* (RM3132–RM589, 0.79–1.38 Mb) and is located adjacent to *BPH32* (cloned at 1.2 Mb) and *BPH29* (cloned at 0.47 Mb). Therefore, *qBPH6* may correspond to *BPH3*, *BPH4*, *BPH25*, *BPH29*, or *BPH32*. Fine mapping is necessary to determine the exact location of *qBPH6* and to clarify its allelic relationship with known BPH resistance genes.

BPH resistance genes have so far been identified on chrs. 1, 3, 4, 6, 7, 8, 9, 10, 11, and 12 across different genetic resources (Yan *et al.* 2023). Here, we detected *qBPH5* between markers RM7029 (0.54 Mb) and RM1024 (1.17 Mb) on the short arm of chr. 5. This region has not been previously reported to contain any BPH resistance genes, so *qBPH5* is a new QTL. Further study such as fine mapping and functional validation will be essential to confirm the exact location and identify the resistance gene(s) at *qBPH5*.

The remaining QTLs—*qBPH2*, *qBPH9*, *qBPH11*, *qBPH12.1*, and *qBPH12.2*—could not be confirmed in the F_2_ populations. We attribute this failure of validation to segregation distortion and weak phenotypic effects. Segregation distortion, whereby allele frequencies deviate from expected Mendelian ratios, can interfere with linkage map accuracy and reduce QTL detection power, especially when tightly linked to a QTL (Zhang *et al.* 2010). In our BRILs, the segregation ratio varied among SSR markers. Chi-squared tests of markers identified as linked to QTLs through single-marker analysis (Table 1) showed that chi-squared values of three markers—RM243, linked to *qBPH1* (χ^2^ = 16.42), RM410, linked to *qBPH9* (χ^2^ = 5.28), and RM247, linked to *qBPH12.1* (χ^2^ = 8.18)—were >3.84 (critical value with df = 1, α = 0.05), indicating segregation distortion, so these QTLs might be false positives.

*qBPH2*, *qBPH11*, and *qBPH12.2* could not be detected in F_2_ populations through QTL analysis, presumably owing to their minor effect in MSST. In addition, the masking effect of major genes on QTLs with minor effects is common in complex traits in rice, such as in resistance- and yield-related traits (Yu *et al.* 2003). The minor effects of *qBPH2* and *qBPH11* are likely masked by the presence of the larger-effect QTLs *qBPH5* and *qBPH6*, respectively. Consequently, our inability to validate *qBPH2*, *qBPH9*, *qBPH11*, *qBPH12.1*, and *qBPH12.2* in the F_2_ populations can be attributed to segregation distortion and their weak phenotypic effects. Confirming these QTLs will require greater population sizes and advanced mapping populations such as near-isogenic lines.

Several studies have shown that highly resistant rice cultivars often carry a combination of minor QTLs in addition to one or more major resistance genes. Such combinations can contribute to more durable resistance (Hu *et al.* 2016). For instance, the Sri Lankan ‘Rathu Heenati’ carries two major genes, *BPH3* and *BPH17*, and several minor QTLs on chrs. 2, 3, 4, 6, and 10, and has strong resistance to all four BPH biotypes present in South-East Asia since the 1970s (Jairin *et al.* 2007, Sun *et al.* 2005). In our study, W0630 showed strong resistance to the current highly virulent population Koshi-2013 (Fig. 1). However, despite the identification of both *qBPH5* and *qBPH6* in W0630, the pyramided line carrying both QTLs did not achieve the same level of resistance as the donor parent. Lines carrying a single QTL had reduced damage scores and BPH settling percentages. Although the line carrying both QTLs had stronger resistance by MSST than either line carrying one QTL, its antixenosis resistance was not as strong as that of W0630 (Fig. 4), so additional minor QTLs may contribute to the robust resistance of W0630.

In the antixenosis test, the pyramided line (*qBPH5* + *qBPH6*) did not show a clear difference from ‘Nipponbare’ (Fig. 4B). In several studies, gene behavior can fluctuate across different genetic backgrounds, as demonstrated in multiple studies, potentially affecting resistance expression (Marcel *et al.* 2008, Palloix *et al.* 2009, Sun *et al.* 2006). In our study, the pyramided line was selected from among the BRILs, it contains other W0630 segments in addition to the QTL regions. Those W0630 segments could interact with the ‘Nipponbare’ genetic background and might result in the susceptibility or variability in antixenosis of the pyramided line. For future studies, advanced pyramided lines carrying only the target QTL regions with minimal donor background could improve the accuracy in evaluating the effect of the pyramided line.

*qBPH1*, *qBPH5*, and *qBPH6* contribute to BPH resistance, and the pyramiding of *qBPH5* and *qBPH6* enhances resistance. Further investigation is needed to identify the precise locations of these QTLs and to explore their potential for use in breeding programs aimed at developing durable BPH-resistant rice cultivars.

## Author Contribution Statement

NHN and DF designed the study. NHN, TI, and DF developed the plant materials. SSM provided BPH populations. SZ supported the research and writing. NHN and DF performed experiments and wrote this paper.

## Supplementary Material

Supplemental Figures

## Figures and Tables

**Table 1.** QTLs for BPH resistance detected in the backcross recombinant inbred lines between *O. sativa* ‘Nipponbare’ and *O. rufipogon* W0630 by single-marker analysis

QTL	Chr.	Marker	Physical location (Mb)	*F*-value	*P*-value	PVE (%)	Additive effect*^a^*
*qBPH1*	1	RM243	7.97	15.08	0.000	8.66	–1.51
*qBPH2*	2	RM263	25.89	14.45	0.000	7.66	–0.61
*qBPH5*	5	RM122	0.28	6.51	0.012	3.93	–0.43
*qBPH6*	6	RM587	2.29	11.29	0.001	7.82	–0.63
*qBPH9*	9	RM410	17.59	11.71	0.001	6.77	–0.64
*qBPH11*	11	RM167	4.06	5.63	0.019	3.42	–0.37
*qBPH12.1*	12	RM247	3.19	18.59	0.000	10.47	–1.03
*qBPH12.2*	12	RM309	21.52	12.03	0.001	6.35	–0.64

*^a^* Additive effect indicates the effect of the allele from *O. rufipogon* W0630.

**Table 2.** QTLs for BPH resistance detected in the backcross recombinant inbred lines between *O. sativa* ‘Nipponbare’ and *O. rufipogon* W0630 by interval mapping and composite interval mapping

QTL	Chr.	Interval mapping						Composite interval mapping				
		Marker interval	Physical location (Mb)	LOD score	PVE (%)	Additive effect*^a^*		Marker interval	Physical location (Mb)	LOD score	PVE (%)	Additive effect*^a^*
*qBPH1*	1	RM243–RM35	7.90–8.41	3.8	19.8	–2.30		RM35–RM580	8.41–9.61	3.9	7.3	–1.40
*qBPH2*	2	RM221–RM6	27.62–29.59	3.2	13.1	–0.69		RM3515–RM263	24.04–25.89	4.6	9.4	–0.64
*qBPH6*	6	RM587–RM204	2.29–3.17	2.6	8.2	–0.69		–	–	–	–	–
*qBPH9*	9	RM566–RM7427	14.65–16.53	3.2	15.6	–1.16		–	–	–	–	–
*qBPH12.1*	12	RM247–RM7619	3.19–4.83	4.0	11.3	–1.04		RM247–RM7619	3.19–4.83	4.4	8.4	–0.96
*qBPH12.2*	12	RM309–RM463	21.52–22.16	2.6	7.7	–0.65		RM309–RM463	21.52–22.16	2.8	5.9	–0.58

*^a^* Additive effect indicates the effect of the allele from *O. rufipogon* W0630.

**Table 3.** QTLs for BPH resistance detected in the F_2_ population between BRIL16 and *O. sativa* ‘Nipponbare’ by composite interval mapping

QTLs	Chr.	Marker	Physical location (Mb)	LOD score	Phenotypic variance (%)	Additive effect*^a^*	Dominant effect*^a^*
*qBPH1*	1	RM23–RM5638	10.71–20.93	3.7	12.8	–0.52	–0.59
*qBPH6*	6	RM589–RM204	1.38–3.17	5.5	17.2	–0.72	0.21

*^a^* Negative effect indicates the effect of the allele from *O. rufipogon* W0630.

**Table 4.** QTLs for BPH resistance detected in the F_2_ population between BRIL29 and *O. sativa* ‘Nipponbare’ by interval mapping

QTL	Chr.	Marker	Physical location (Mb)	LOD score	Phenotypic variance (%)	Additive effect*^a^*	Dominant effect*^a^*
*qBPH5*	5	RM7029–RM1024	0.54–1.17	3.68	11.74	–0.54	0.03

*^a^* Negative effect indicates the effect of the allele from *O. rufipogon* W0630.
