# Supplementary material for: Identification and characterization of QTLs for brown planthopper resistance from wild rice, Oryza rufipogon
Source: Breed Sci. 2025 Nov 21;75(5):455–62. doi: 10.1270/jsbbs.25027 (PMC13148877; doi:10.1270/jsbbs.25027)
Supplement: Supplementary file 1 — Supplemental Figures [file 75_455_s1.pdf]

doi: 10.1270/jsbbs.25027

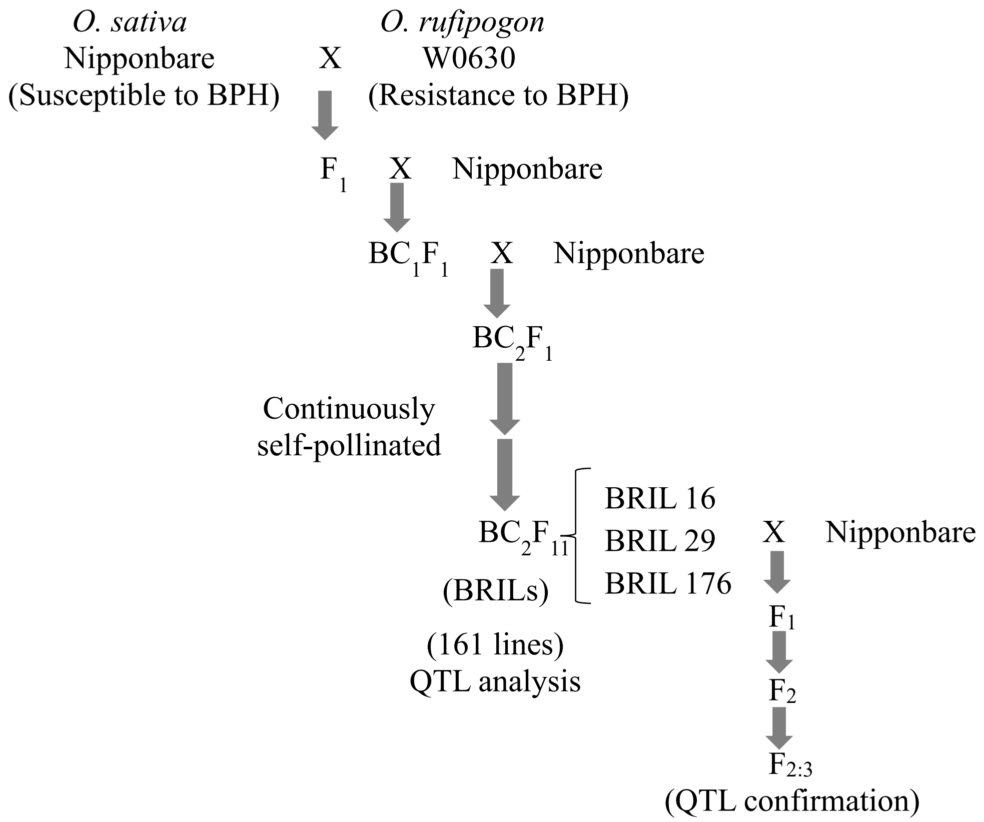

Supplemental Fig. 1. Breeding scheme for the development of backcross recombinant inbred lines (BRILs) (Thanh *et al*. 2011) and segregating population for QTL confirmation.

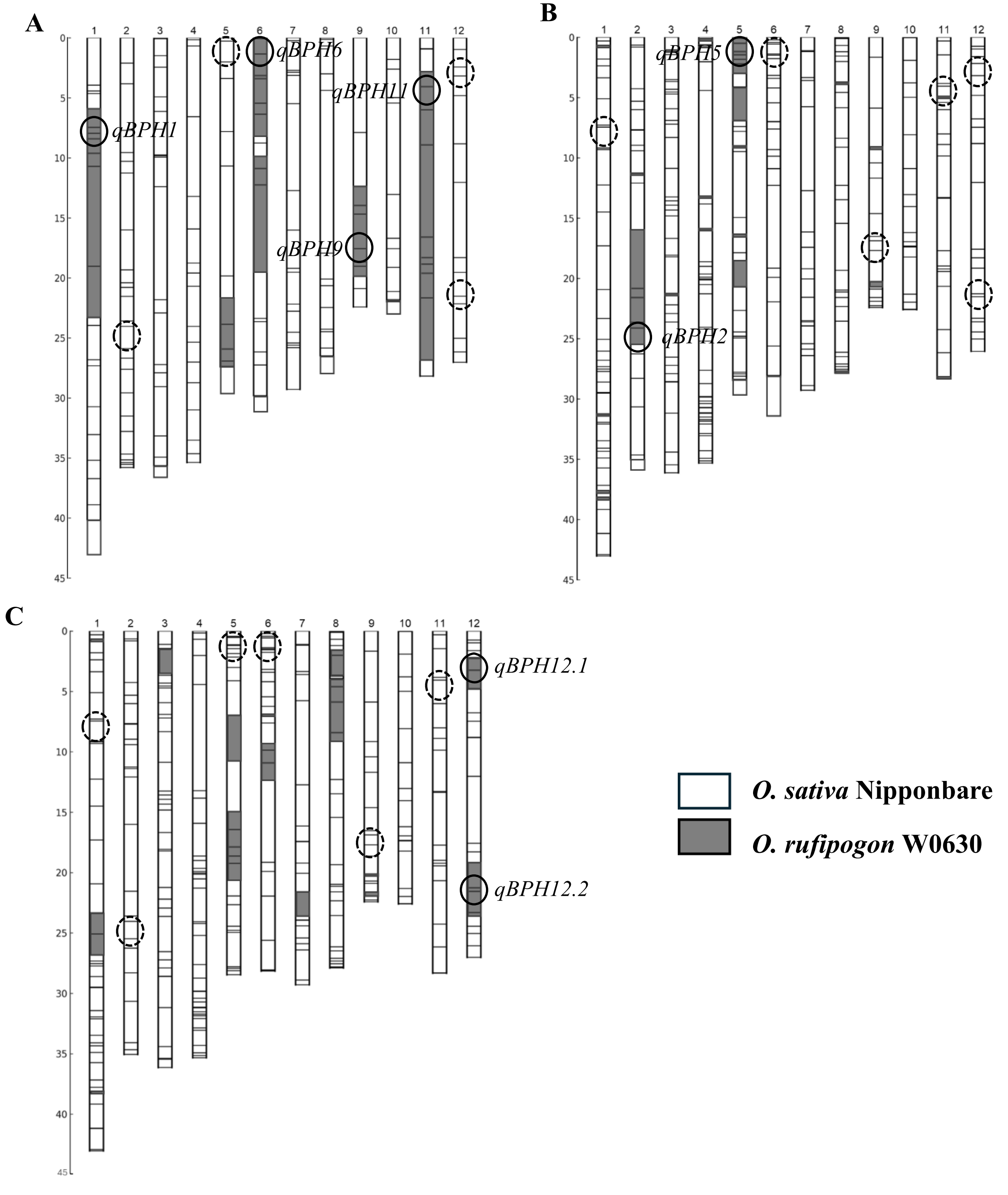

Supplemental Fig. 2. Graphical genotype of BRIL16 (A), BRIL29 (B) and BRIL176 (C). The 12 bars indicate 12 chromosomes of rice. Horizontal lines across the chromosomes indicate the positions of polymorphic SSR markers.

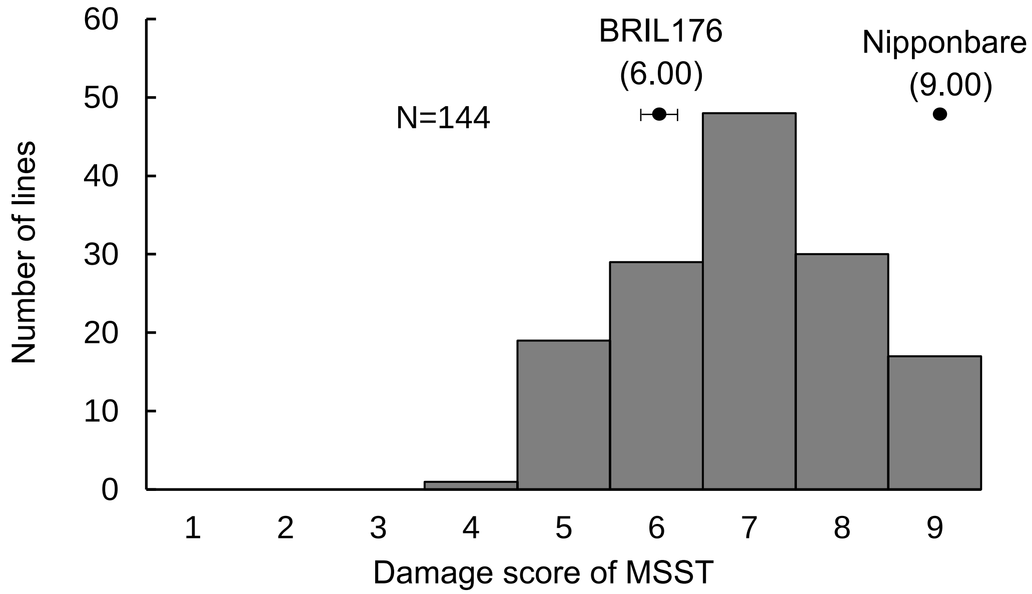

Supplemental Fig. 3. Frequency distribution of damage score of modified seedbox screening test (MSST) in the $F_{2:3}$ population derived from a cross between BRIL176 Nipponbare by Hadano-66.
